# Job satisfaction and its modeling among township health center employees: a quantitative study in poor rural China

**DOI:** 10.1186/1472-6963-10-115

**Published:** 2010-05-10

**Authors:** Jun A Liu, Qi Wang, Zu X Lu

**Affiliations:** 1 Department of Social Medicine, School of Public Health, Tongji Medical College, Huazhong University of Science and Technology, No.13, Hangkong Road, Wuhan, Hubei Province, PR China; 2 Department of Epidemiology and Biostatistics, School of Public Health, Tongji Medical College, Huazhong University of Science and Technology, No.13, Hangkong Road, Wuhan, Hubei Province, PR China

## Abstract

**Background:**

Job satisfaction is important to staff management of township health centers (THCs), as it is associated with organizational performance, quality of care and employee retention. The purpose of this study was to measure job satisfaction level of THC employees in poor rural China and to identify relevant features in order to provide policy advice on human resource development of health service institutions in poor regions.

**Methods:**

A self-completion questionnaire was used to assess the job satisfaction and relevant features (response rate: 90.5%) among 172 employees (i.e., clinic doctors, medico-technical workers and public health workers) of 17 THCs in Anhui and Xinjiang provinces of China. The study covered a time period of two months in 2007.

**Results:**

The mean staff job satisfaction scored 83.3, which was in the category of "somewhat satisfied" on a scale ranging from 0 (extremely dissatisfied) to 100 (extremely satisfied) by employing Likert's transformation formula. Exploratory factor analysis (EFA) revealed eight domains involved in modeling of job satisfaction, among which, the caregivers were more satisfied with job significance (88.2), job competency (87.9) and teamwork (87.7), as compared with work reward (72.9) and working conditions (79.7). Mean job satisfaction in Xinjiang (89.7) was higher than that in Anhui (75.5).

**Conclusions:**

Employees of THCs have moderate job satisfactions in poor areas, which need to be raised further by improving their working conditions and reward.

## Background

Job satisfaction is defined as the positive personal perception towards work or work experiences [1]. In fact, job satisfaction has been identified as an important determinant of employee retention, turnover and work performance [2]. In health service sectors, job satisfaction is highly associated with staff's intention to quit, quality and efficiency of services, and patient satisfaction [3]. It has been reported that doctors with higher job satisfaction are more likely to provide more satisfactory services and produce better therapeutic effect than those with the lower one [4]. Therefore, higher job satisfaction tends to result in much higher patient satisfaction and reduce medical costs, thereby making a hospital more competitive [5].

Recently, doctor dissatisfaction has become a subject of keen investigation [6]. In 2001, Richard Smith posed a question "Why are doctors so unhappy?" in BMJ, believing that the reasons varied, some of which were deep and complicated [7]. So far, health service institutions in most countries introduced a patient-centered management, which, to some extent, hurt the interest of doctors [8]. Moreover, in 2000, WHO designed a measure of responsiveness to assess the respect of consumers' rights [9]. These moves, unknowingly, erode the rights of doctors, and impair their motivation to better serve patients.

In China, township health centers (THCs) are primary health care organizations, owned by the state or collectives, and provide public health services and primary medical services to above 800 million populations in rural China, which play important roles in the three-tier rural health-care network and the "New Rural Corporative Medical System" [10]. However, job satisfaction and retention of THC employees are lower than those of employees working in urban community health centers (CHCs) in China. The Guangdong province is among the richest regions of China, but investigations have shown that job satisfaction of the THC staff in the province was not high, in that caregivers there were not satisfied with their work reward and professional development [11]. Short of long-term governmental investment, some THCs are struggling to survive, especially those in poverty-hit rural areas, which hardly meet the healthcare needs of the local population [12].

To help THCs provide better health services in poor rural China, Chinese Ministry of Health and Hong Kong Kadoorie Foundation elected to launch a Rural Community Health Promotion Project in 2004. One major purpose of the project was to improve overall conditions of THCs, including site construction, provision of basic medical equipment and skill training. In 2005, the project was started in 17 THCs in poor regions of Xinjiang and Anhui. This article was to assess staff job satisfaction of THCs in the poor regions covered by the project and identify critical determinants to job satisfaction, with an attempt to work out strategies to improve the job satisfaction and retention in these institutions.

## Methods

### Overview

**Figure 1:**
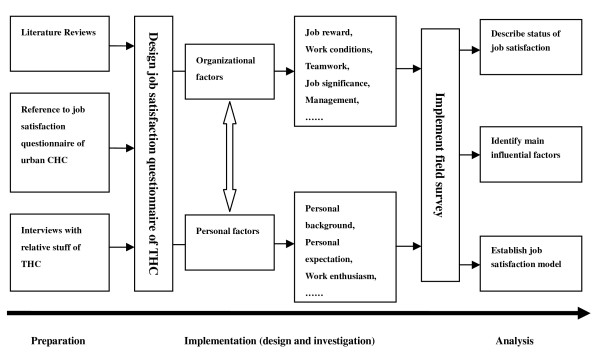
**Research framework of job satisfaction among THC employees**.

The framework of our research is shown in Figure 1. In preparation phase, reports on job satisfaction [13-16] were reviewed and a questionnaire on job satisfaction of THC employees was developed on the basis of both organizational and individual features. The self-completed questionnaire was employed to estimate the job satisfaction of the employees of THCs covered by Kadoorie Project. Quantitative analyses were applied to describe the staff job satisfaction status and identify critical influencing factors and to establish a model of job satisfaction.

In this study, all subjects worked in the fields of clinical, medico-technical, and public health services of 17 THCs, 7 in Anhui and 10 in Xinjiang. Administrators were not included. All the THCs were low-level non-profit medical institutions located in poor rural areas. Seven THCs in Anhui Province were financially independent, and the other 10 institutions in Xinjiang were fully supported by local governments. All the subjects were asked to complete the questionnaire anonymously. Investigators, if necessary, would provide assistance in filling out the questionnaire. Questionnaires took, on average, two hours to fill out in each THC. In the survey, some subjects were too busy with their work to fill out the questionnaires. In the end, a total of 172 employees completed the questionnaires: a response rate of 90.5%.

In order to collect relevant information from respondents, a study design based on epidemiological investigations and face-to-face interviews was conducted by using self-maid questionnaires without any experimental procedures on humans or animals. We promised keeping secret of personal information of all subjects. And all questionnaires were filled anonymously. Therefore this study did not harm the ethics of subjects. All subjects gave their informed consent prior to the study.

### Questionnaire

Various instruments are used to evaluate job satisfaction [17], among which the job description index (JDI) [16,18] and the job satisfaction scale (JSS) [19] are most popular. These instruments, however, are not suitable for assessing job satisfaction of medical or health workers in China. Moreover, no questionnaire is available for the evaluation of job satisfaction of THC workers in rural China. Therefore, on the basis of an extensive literature review, interviews with THC doctors, a careful study of the questionnaire for Chinese urban CHC [13,14], we developed a questionnaire for evaluating job satisfaction of Chinese THC employees.

The job satisfaction instrument for Chinese urban CHCs included 35 items and was previously used for measuring job satisfaction among GPs [13]. To suit THC staff, the instrument was revised and tailored to the features of THC workers. The job satisfaction questionnaire for THC workers contained only 28 items, covering working conditions, remuneration, cooperation with colleagues, job significance, management and so on (Table 1), measured on a 5-point scale ranging from 1 (extremely dissatisfied) to 5 (extremely satisfied). The instrument also included questions on personal information of THC employees such as age, gender, education background, professional title etc. The items were so arranged that halo effect was avoided.

**Table 1:** Variables involved in each principal factor and their factor loadings

Common factors	Variables	Loadings
Factor 1	Overall work condition	0.755
Work conditions and atmosphere (5 items)	Space of office	0.693
	Leadership improves work climate	0.637
	Development improves work condition	0.627
	Staff adscription from improvement of work condition	0.512

Factor 2	Income from salary	0.786
Job reward (4 items)	Distribution of rewards	0.769
	Welfare and treatment	0.758
	Possibilities of promotion	0.444

Factor 3	Suggestions being adopted	0.693
Work achievements (5 items)	Abilities fully to play	0.565
	Busy at work	0.546
	Achievement from work	0.555
	Cohesion from work	0.529

Factor 4	Competency of knowledge	0.857
Job competency (3 items)	Competency of skills	0.793
	Competency being recognized by coworkers	0.590

Factor 5	Character of job being self-recognized	0.704
Job significance (4 items)	Character of job being recognized by leaders	0.702
	Character of job being recognized by coworkers	0.700
	Performance of job being recognized by leaders	0.502

Factor 6	Cooperation with coworkers	0.747
Team work (3 items)	Communication with coworkers	0.685
	Relationship between coworkers	0.579

Factor 7	Management is helpful for development of organization	0.887
Management (2 items)	Ability of management needs being improved further	0.869

Factor 8	Concern for development of organization Positive feeling for their work	0.802 0.567
Work enthusiasm (2 items)		

Note: Principal components with equamax rotation.

### Data analysis

#### Standard 5-point Likert-type scale

Staff job satisfaction is typically rated on a 5-point Likert scale from 1 (extremely dissatisfied) to 5 (extremely satisfied). And the standard satisfaction scale ranging from 1 to 5 were converted into a 0-to-100 scale by utilizing a formula as below.

Where "adjSS" and "stdSS" are "adjusted satisfaction score" and "standard satisfaction score" respectively.

With the new scoring method, job satisfaction fell into 5 categories: "extremely dissatisfied" (adjSS: 10-29), "dissatisfied" (30-49), "generally satisfied or not" (50-59), "satisfied" (70-89), and "extremely satisfied" (90-100).

#### Exploratory factor analysis (EFA)

Correlation among the 28 items were found to be weak, and, with a threshold set at 0.8 [20], they were not collinear with each other. Also, regression analysis revealed no multicollinearity. EFA was performed to identify common factors that can cover all the aspects of job satisfaction of the respondents by using principal component analysis and equamax rotation. Eight common factors were extracted, with cumulative initial eigenvalue being 77.88%. Original satisfaction score multiplied by corresponding factor loading equals principal factor scores. The Cronbach's alpha reliability coefficient was 0.851 for entire set of items.

#### Linear regression analysis

Stepwise multi-factor linear regression was performed to analyze dependence of staff's total job satisfaction on independent variables as area, gender, age, race, education background, professional title, job type, tenure, pay and the 8 principal factors. In linear regression model, categorical variables were converted to dichotomous dummy variables. Since race and age were respectively related with area and tenure, only one of the two related variables was used at a time in regression modeling to avoid multicollinearity. The *P *value considered statistically significant was P < 0.05. The linear regression model was statistically significant and determination coefficient (R square) was 0.642, suggesting that the model, to some extent, could interpret the variations of overall job satisfactions of the subjects.

## Results

### Demographic Characteristics

In this study, 172 THC employees, including 66 men (38.4%) and 106 (61.6%) women, took part in the investigation, accounting for 80% of the all health workers (The administrative personnel and employees engaged in logistical services were excluded). Of all the subjects, 77 (44.8%) were from Anhui and 95 (55.2%) from Xinjiang. All the Anhui THC workers were Hans, and the Xinjiang subjects were Uighurs. The participants worked in clinical (N = 75, 43.6%), medico-technical (N = 52, 30.2%) and nursing (N = 36, 20.9%) and public health (N = 9, 5.3%) fields. As to education background, 59 (34.3%) had a bachelor's degree, 107 (62.2%) received secondary technical education and 6 (3.5%) did not have any background of medical education. 62.2% of the respondents were junior professionals. 58.2% of the subjects received a monthly salary of no more than 1000 RMB, but the mean monthly salary (1150 RMB) of Xinjiang THC employees was higher than that (605 RMB) of their Anhui counterparts.

### Common factors of job satisfaction

During EFA, each item in the domains had large factor loadings, ranging from 0.444 to 0.887 (≧0.44). On the basis of our empirical judgment, the 28 items were categorized into 8 domains (Table 1): The first factor relating to work conditions and atmosphere included 5 items; the second factor concerning job reward involved 4 items; the third factor regarding personal achievements had 5 items; the fourth one about job competency consisted of 3 items; the fifth one relating to job significance contained 4 items; the sixth one about team work included 3 items; the seventh factor about management had 2 items; and the eighth factor concerning work enthusiasm contained 2 items.

### Job satisfaction scores

The scores of job satisfaction of all the subjects are listed in Table 2. Arithmetic mean of job satisfaction score of all the medical staff was 83.3, equivalent to "somewhat satisfied" on Likert's scale. The average job satisfaction score of the Xinjiang THC staff was 89.7 while that of their Anhui counterparts was 75.5, suggesting job satisfaction was higher in Xinjiang THC workers than in Anhui subjects. Scores reflecting job significance, job competency and team work were highest, with their arithmetic means being 88.2, 89.7 and 87.7 respectively. Work conditions and atmosphere and job reward were lowest, with corresponding average score being 79.7 and 72.2.

**Table 2:** Job satisfaction scores in 8 job satisfaction domains

Variables	Anhui's THCs mean score (SD)	Xinjiang's THCs mean score(SD)	Total score
Job significance	82.6 (12.0)	92.7 (10.0)	88.2
Job competency	83.8 (11.2)	91.3 (9.4)	87.9
Team work	81.9 (10.6)	92.4 (9.3)	87.7
Work enthusiasm	78.2 (15.6)	90.9 (9.6)	85.2
Management	78.3 (14.0)	88.8 (11.9)	84.1
Work achievements	73.4 (16.3)	87.5 (11.7)	81.2
Work conditions and atmosphere	71.6 (19.1)	86.3 (15.9)	79.7
Job reward	54.2 (21.1)	86.8 (12.9)	72.2

Total score	75.5	89.7	83.3

### Demographic characteristics associated with job satisfaction

Table 3 shows that age, gender, race, professional title, service time and salary had significant influence on job satisfaction (P < 0.01), while job type and educational level did not (P > 0.05). The average score of job satisfaction in workers 40-49 years old were lower than that of the others (P < 0.01); the score of males was lower than that of females (P < 0.01); the score of Hans was lower than that Uighurs (P < 0.01); the score of the medical workers without professional titles and with a doctor or above professional titles was lower than those with the other titles (P < 0.01); the score of employees working for 16-30 years was lower than the others (P < 0.05); the score of subjects with monthly salary between 500-800 RMB was lower than the others (P < 0.01).

**Table 3:** Demographic characteristics associated with staff job satisfaction

Variables	Groups	N	Score	P
Age (years old)	No more than 30	66	87.3	0.002**
	30-39	77	81.1	
	40-49	22	78.0	
	50-60	7	86.5	
Gender	Male	66	79.5	0.001**
	Female	106	85.7	
Race	Han	84	76.3	<0.001**
	Uighur	88	90.0	
Job type	Clinic	75	82.0	0.101
	Nursing	52	86.5	
	Medico-technique	36	82.7	
	Other else	9	78.4	
Professional title	Doctor in charge or above	18	79.7	0.001**
	Doctor	55	79.9	
	Assistant doctor	52	84.5	
	Primary health worker	37	89.7	
	No technical title	10	78.6	
Educational background	Bachelor degree holders	6	88.7	0.090
	Junior college graduates	53	79.6	
	Technical secondary school graduates	107	84.9	
	No medical educational background	6	80.8	
Tenure (years)	1-5	31	89.2	0.011*
	6-10	56	82.6	
	11-15	39	82.6	
	16-20	26	78.9	
	21-30	13	79.9	
	Over 30	7	89.5	
Salary (RMB per month)	No more than 500	24	85.5	<0.001**
	500-799	40	76.9	
	800-999	36	81.6	
	1000-1499	49	86.1	
	Over 1499	23	89.0	

*p < 0.05 **p < 0.01

### Linear regression model

Table 4 presents 10 independent variables in the final multi-factor linear regression model, including race, educational background and all the 8 principal factors contributing to job satisfaction. In the model, standardized partial regression coefficients of work conditions, reward and cooperation with colleagues were greater than the coefficients of the others. In addition, educational background was found to be significantly associated with job satisfaction (P < 0.05). Employees with a bachelor's degree were less satisfied with their work than employees with no medical education (serving as the control) when the other variables were controlled. And job satisfaction of Uighur employees (from THCs in Xinjiang), with other variables controlled, was 16.7% higher than that of Han workers (from THCs in Anhui).

**Table 4:** Results of multi-factor linear regression analysis

Relative factors	Partial regression coefficients	Standard error	Standardized partial regression coefficients	T	P
Constant	4.692				
Work conditions and atmosphere	0.261	0.028	0.463	9.408	<0.001
Job reward	0.228	0.033	0.406	6.922	<0.001
Work achievements	0.131	0.027	0.232	4.773	<0.001
Job competency	0.054	0.027	0.096	2.024	0.045
Job significance	0.151	0.028	0.269	5.410	<0.001
Team work	0.173	0.030	0.308	5.755	<0.001
Management	0.152	0.027	0.271	5.580	<0.001
Work enthusiasm	0.163	0.028	0.290	5.907	<0.001
Race(Uighur)	0.188	0.076	0.167	2.466	0.015
Education (Bachelor's degree)	-0.130	0.060	-0.107	-2.159	0.032

R^2 ^= 0.642, adjR^2 ^= 0.617 (F = 26.060, p < 0.001)

## Discussion

Staff job satisfaction in THCs has important implications for sustainable development of basic healthcare in China, but so far health decision-makers failed to pay enough attention to job satisfaction of grassroots medical workers. Moreover, they have little knowledge about factors that are associated with job satisfaction and dissatisfaction [21]. Past experience showed that the THCs can not survive and thrive without a team of dedicated workers equipped with adequate medical skills [11]. As primary health service providers, THCs need favorable work environment and conditions to operate efficiently and to meet rational demands of their employees.

According to previous reports, job satisfaction is associated with a wide array of factors, including organizational factors such as job reward and work conditions and personal factors like sense of work achievements and work enthusiasm [22-24]. In our study, exploratory factor analysis was conducted and 8 common factors were identified, which covered virtually all job satisfaction-related factors, such as work conditions, job reward, work achievements, work competency, work significance, team work, management, and work enthusiasm. And compared with JDI that contains the 5 domains (work, compensation, promotion opportunities, superiors, and co-workers) [25], our self-designed questionnaire was more comprehensive, and suitable for the Chinese medical employees working at poverty-stricken rural area in evaluating job satisfactions. Our results showed that the job satisfaction model in this study had greater explanatory power (*R*^2 ^= 0.642) and was more reliable (the Cronbach's alpha reliability coefficient was 0.851). Our study provides a good starting point for further development of standard scale for measuring job satisfaction of workers engaged in THC services in poor rural China.

In the study, we found that the mean score of job satisfaction of workers covered by Kadoorie project was 83.3, which was at "somewhat satisfied" level on Likert's scale. We also found that most staff considered their job to be of importance and got along well with their fellow workers. What they felt most dissatisfied with were work reward (i.e. welfare, pay, and promotion opportunity), working conditions, and sense of work achievements [22]. In the field survey, we noticed that some caregivers complained that salary was too low, considering their experience and skill levels, which substantially hurts their work enthusiasm. Other related studies demonstrated that medical workers were more satisfied with their work and team, while less satisfied with job reward and opportunities of professional promotion [25,26]. Compared to urban medical institutions, grassroots heath service units have poor work conditions and fewer promotion opportunities. Therefore, inability to realize personal value has been a major concern of medical workers at THC in poor rural China.

THC employees had higher job satisfaction in Xinjiang than in Anhui. This might be due to relatively higher level of job reward THC that the employees in Xinjiang could receive. Actually, since Xinjiang province was a frontier region located in northwest China, where most residents were Uighurs, the national government invested a lot to support rural health services in the region and THC employees there receive more than a monthly salary of 1000 RMB, approximately twice that received by THC employees in Anhui. At present, for health workers in poor rural areas, salary has become a critical factor that influences job satisfaction of THC employees. Herzberg [27] believed that pay, work conditions, job security and relation with colleagues are essential factors that dictate job satisfaction. And failure to meet the demands of employees in these regards will lead to complaints. Moreover, job satisfaction was found to be inversely related with turnover of employees [28], i.e., poor job satisfaction is linked to high turnover [16,29]. If most employees, especially backbone ones, in a THC intend to quit, the result will be disastrous. At present, high turnover among high-quality doctors engaged in THC has been a major threat to the survival of THCs in the rural China [30], which needs to be addressed immediately.

Personal factors, such as age, gender and race also impact on job satisfaction. According to previous reports, females tend to be more satisfied with their job than males [26], and a U-shaped relation was observed between age and job satisfaction [31]. Our results were consistent with these findings. Males, middle-aged and higher professional title holders in our series were least satisfied with their job. However, these caregivers are main force of THCs and care more about their work environment and professional development. Therefore, policy-makers and THC supervisors should address their demands and concerns to bring their talents into full play.

At present, it is necessary to design a well-tailored job satisfaction instrument for Chinese medical staff working in impoverished regions. This study is a preliminary effort to achieve the goal. Compared with the JDI instrument [25], which has 14 items, and the JSS instrument [6,19] that consists of 10 items, our self-designed questionnaire is relatively more complicated and time-consuming, which may pose a problem with busy medical staff. Additionally, because the sample size of our study was comparatively small, studies of large cohorts need to be conducted to further revise and perfect this questionnaire.

The response rate (90.5%) in our study was higher than that (81.2%) reported by Yin *et al*. [14], that (73%) by Magne *et al*. [6] and that (75%) by Nicholas *et al*. [25]. This might be because the supervisors of the THCs under Kadoorie Project attached great importance to the investigation. Assessing staff job satisfaction was an important part of the project evaluation, the supervisors asked their employees to fully cooperate with the investigators. And the investigators provided guidance when the subjects had any problems with the completion of the questionnaire.

However, there are still some limitations with the research. The project covered only 17 THCs in two provinces; the sample size was comparatively small, which might, to some extent, affect the stability of multi-factorial analysis. Moreover, this investigation was only a cross-sectional study on the results of the project and had no baseline data for comparison. This more or less impairs the power of our study. Care should be exercised in the interpretation and extrapolation of our findings.

## Conclusions

In conclusions, THC employees have moderate level of job satisfaction in poor areas covered by Kadoorie Project. THC employees are more satisfied with the work significance and cooperation with colleagues, while less satisfied with work conditions, reward and promotion opportunities. To enhance staff job satisfaction, THC supervisors should take measures to improve work conditions, raise work reward and pay more attention to the professional development of their employees.

## Competing interests

The authors declare that they have no competing interests.

## Authors' contributions

JAL and ZXL jointly conceived the ideas for the study. JAL was responsible for the data collection in study fields. QW took care of the data analysis and all authors took part in the result interpretation. JAL prepared manuscript. All authors were involved in the revision of the paper and approved the final version.

## Pre-publication history

The pre-publication history for this paper can be accessed here:

http://www.biomedcentral.com/1472-6963/10/115/prepub
